# Evaluating Mono- and Combination Therapy of Meropenem and Amikacin against Pseudomonas aeruginosa Bacteremia in the Hollow-Fiber Infection Model

**DOI:** 10.1128/spectrum.00525-22

**Published:** 2022-04-20

**Authors:** Minyon L. Avent, Kate L. McCarthy, Fekade B. Sime, Saiyuri Naicker, Aaron J. Heffernan, Steven C. Wallis, David L. Paterson, Jason A. Roberts

**Affiliations:** a The University of Queensland, UQ Centre for Clinical Research, Herston, Queensland, Australia; b Queensland Statewide Antimicrobial Stewardship Program, Royal Brisbane and Women’s Hospital, Herston, Queensland, Australia; c Department of Infectious Diseases, Royal Brisbane and Women’s Hospital, Brisbane, Australia; d School of Medicine, Griffith University, Southport, Queensland, Australia; e Department of Pharmacy, Royal Brisbane and Women’s Hospital, Brisbane, Australia; f Department of Intensive Care Medicine, Royal Brisbane and Women’s Hospital, Brisbane, Australia; g Division of Anaesthesiology, Critical Care Emergency and Pain Medicine, Nîmes University Hospital, University of Montpellier, Nîmes, France; University of Guelph

**Keywords:** amikacin, meropenem, *Pseudomonas aeruginosa*, pharmacodynamic, hollow fiber infection model

## Abstract

Debate continues as to the role of combination antibiotic therapy for the management of Pseudomonas aeruginosa infections. We studied the extent of bacterial killing by and the emergence of resistance to meropenem and amikacin as monotherapies and as a combination therapy against susceptible and resistant P. aeruginosa isolates from bacteremic patients using the dynamic *in vitro* hollow-fiber infection model. Three P. aeruginosa isolates (meropenem MICs of 0.125, 0.25, and 64 mg/L) were used, simulating bacteremia with an initial inoculum of ~1 × 10^5^ CFU/mL and the expected pharmacokinetics of meropenem and amikacin in critically ill patients. For isolates susceptible to amikacin and meropenem (isolates 1 and 2), the extent of bacterial killing was increased with the combination regimen compared with the killing by monotherapy of either antibiotic. Both the combination and meropenem monotherapy were able to sustain bacterial killing throughout the 7-day treatment course, whereas regrowth of bacteria occurred with amikacin monotherapy after 12 h. For the meropenem-resistant P. aeruginosa isolate (isolate 3), only the combination regimen demonstrated bacterial killing. Given that tailored antibiotic regimens can maximize potential synergy against some isolates, future studies should explore the benefit of combination therapy against resistant P. aeruginosa.

**IMPORTANCE** Current guidelines recommend that aminoglycosides should be used in combination with β-lactam antibiotics as initial empirical therapy for serious infections, and otherwise, patients should receive β-lactam antibiotic monotherapy. Given the challenges associated with studying the clinical effect of different antibiotic strategies on patient outcomes, useful data for subsequent informed clinical testing can be obtained from *in vitro* models like the hollow-fiber infection model (HFIM). Based on the findings of our HFIM, we propose that the initial use of combination therapy with meropenem and amikacin provides some bacterial killing against carbapenem-resistant P. aeruginosa isolates. For susceptible isolates, combination therapy may only be of benefit in specific patient populations, such as critically ill or immunocompromised patients. Therefore, clinicians may want to consider using the combination therapy for the initial management and ceasing the aminoglycosides once antibiotic susceptibility results have been obtained.

## INTRODUCTION

Pseudomonas aeruginosa is the Gram-negative bacterium most commonly associated with mortality and morbidity in hospitalized and immunocompromised individuals (1).

Severe P. aeruginosa infections, including bacteremia, require optimized antimicrobial management, given mortality rates of up to 61% (2). Debate currently exists as to whether combination therapy can result in better outcomes in the management of severe P. aeruginosa infections. Arguments that support the use of combination therapy have done so on the grounds of *in vitro* synergy, an increased likelihood of microbiologically adequate empirical therapy, and the potential to prevent the development of resistance (3). Observational studies suggest that combination drug regimens are advantageous in those individuals with neutropenia and shock (4, 5). In contrast, meta-analyses performed to date have not demonstrated combination therapy to be more effective than monotherapy in the treatment of P. aeruginosa infections (6–8).

Aminoglycosides are most often used in combination with β-lactam antibiotics as initial empirical therapy for serious infections but are not considered appropriate as monotherapy, as they have demonstrated inferiority in clinical studies, including higher mortality rates, compared to β-lactam antibiotics (9–11). These recommendations have been included in the European Committee on Antimicrobial Susceptibility Testing (EUCAST) guidelines (12).

Achieving target pharmacokinetic and pharmacodynamic indices early during the antibiotic course of therapy is associated with an improved patient response and reduced mortality, particularly in serious bloodstream infections (13–15). Wong et al. evaluated pooled data from 98 critically ill patients with monomicrobial Gram-negative bacillary bacteremia who were treated with β-lactam antibiotics; the study identified improved rates of clinical outcome, as defined by completion of the treatment course without changes to antibiotic therapy, when a free β-lactam antibiotic minimum concentration-to-MIC (*fC*_min_/MIC) ratio greater than 1.3 was achieved (16).

Given the challenges of clinically studying the effects of different antibiotic strategies on patient outcome, useful data for subsequent informed clinical testing can be obtained from *in vitro* models (17). The hollow-fiber infection model (HFIM) can simulate the time course of antibiotic concentrations with a specific elimination half-life at a predetermined inoculum over clinically relevant durations, both of which are technically difficult with animal *in vivo* models. The results from the HFIM are also well correlated with clinical endpoints for bacterial killing and time course of emergence of resistance (18). Here, we studied meropenem and amikacin as mono- and combination therapies against susceptible and resistant P. aeruginosa isolates from bacteremic patients and compared the extent of bacterial killing and suppression of resistance (19).

## RESULTS

### *In vitro* susceptibility and mutant frequency studies.

The MICs of meropenem and amikacin for the P. aeruginosa isolates are summarized in Table 1. One isolate was resistant to meropenem with an MIC of 64 mg/L (EUCAST breakpoints are susceptible, ≤2mg/L, and resistant, >8 mg/L). The mutant frequencies for the P. aeruginosa isolates in the presence of amikacin (32 mg/L) were 2.93 × 10^−8^ CFU/mL, 3.21 × 10^−8^ CFU/mL, and 8.94 × 10^−8^ CFU/mL, respectively, for isolates 1 to 3. The mutant frequencies for the P. aeruginosa isolates grown on meropenem-impregnated, cation-adjusted Mueller-Hinton (CaMH) agar (16 mg/L) were <4.10 × 10^−9^ CFU/mL and <5.30 × 10^−9^ CFU/mL for isolates 1 and 2, respectively. For isolate 3, the mutant frequency was 7.42 × 10^−7^ CFU/mL when grown on meropenem-impregnated CaMH agar (256 mg/L). The meropenem MIC following exposure to meropenem-impregnated agar (4-fold the baseline MIC for each isolate) for isolate 3 was 512 mg/L, while no growth was observed for isolates 1 and 2.

**TABLE 1:** Summary of MICs of meropenem and amikacin for the P. aeruginosa isolates

Isolate	Result for^*a*^:			
	Meropenem		Amikacin	
	MIC (mg/L)	Susceptibility	MIC (mg/L)	Susceptibility
1	0.25	S	2	S
2	0.125	S	2	S
3	64	R	4	S

a S, susceptible; R, resistant.

### Pharmacokinetic/pharmacodynamic parameters.

The observed meropenem time above the MIC (*T*_>MIC_) was 100% for susceptible isolates (isolates 1 and 2) for both dosing regimens (1 g every 8 h and 2 g every 8 h) during the first dosing interval and approximately 3% for the resistant isolate (isolate 3) for the 2 g every 8 h regimen. The free β-lactam antibiotic maximum concentration-to-MIC (*fC*_max_/MIC) ratios of amikacin were between 8.4 and 11.7 for isolates with an MIC of 2 mg/L and between 3.5 and 4.1 for the isolate with an MIC of 4 mg/L.

### Hollow-fiber infection model.

**FIG 1:**
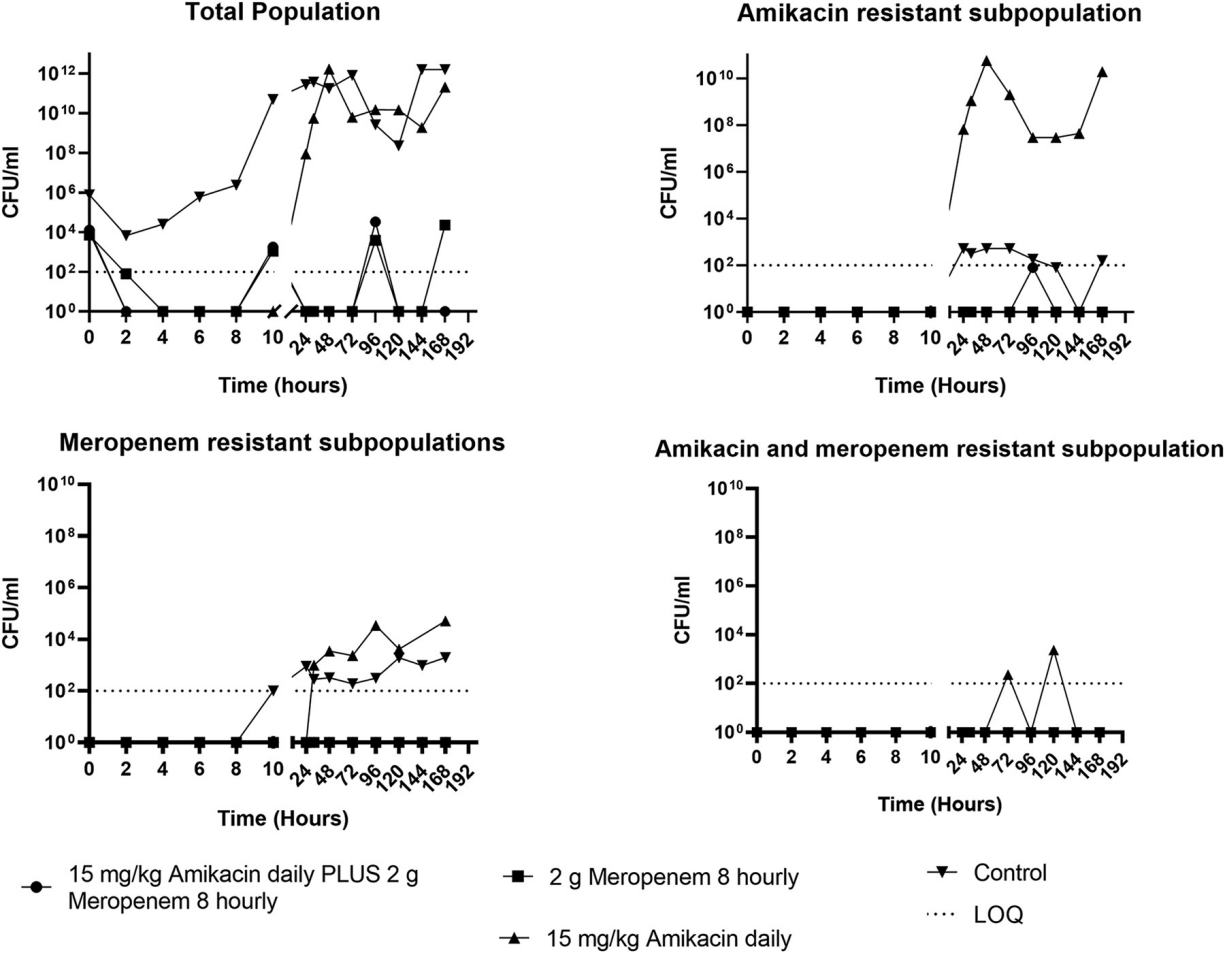
The effect of amikacin or meropenem monotherapy versus amikacin/meropenem combination therapy on the bacterial density of a P. aeruginosa isolate (isolate 1, susceptible P. aeruginosa isolate) in a hollow-fiber infection model. LOQ, limit of quantitation.

**FIG 2:**
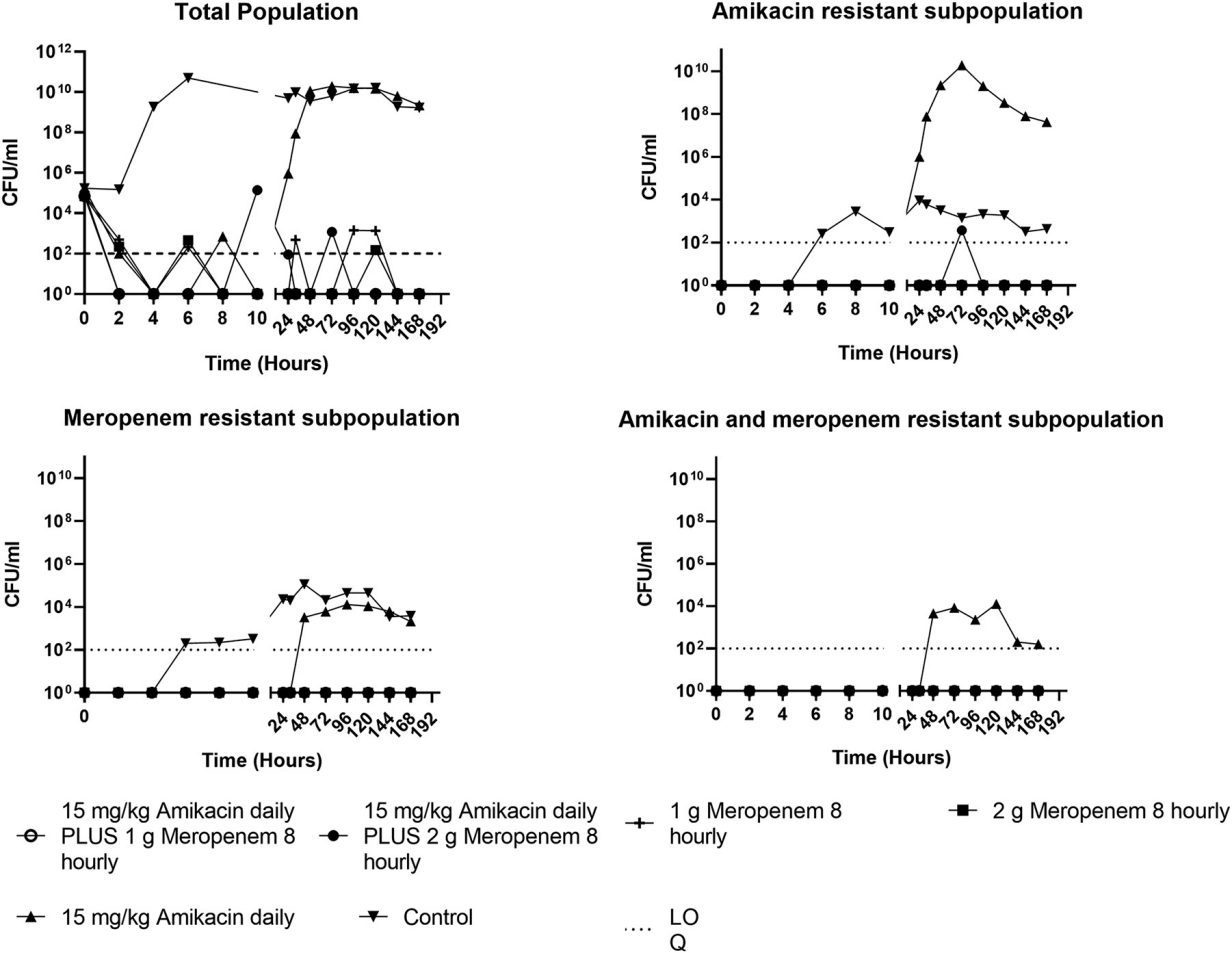
The effect of amikacin or meropenem monotherapy versus amikacin/meropenem combination therapy on the bacterial density of a P. aeruginosa isolate (isolate 2, susceptible P. aeruginosa) in a hollow-fiber infection model. LOQ, limit of quantitation.

For isolates susceptible to both amikacin and meropenem (isolates 1 and 2), monotherapy with either drug rapidly reduced the bacterial density by ~5 log_10_ CFU/mL within 4 h of initial dosing (Fig. 1 and 2). This was comparable with the results for the combination regimens, although the combination achieved the same bacterial killing within 2 h (Fig. 1 and 2). In addition, the combination regimen and meropenem monotherapy were both able to sustain bacterial killing throughout the 7-day experiment duration. Conversely, amikacin monotherapy was only able to sustain bacterial killing for the first 12 h of treatment for all isolates (Fig. 1 and 2).

Bacterial regrowth on standard CaMH agar mirrored that on amikacin-containing CaMH agar following exposure to amikacin monotherapy (Fig. 1 and 2). Resistance to amikacin was confirmed with MIC increases from 2 mg/L (Table 1) to 64 mg/L and 32 mg/L, respectively, for isolates 1 and 2 (Tables 2 and 3). Monotherapy with meropenem did not result in the amplification of growth of subpopulations resistant to either amikacin or meropenem. Treatment with meropenem alone or when combined with amikacin suppressed the growth of a meropenem-resistant subpopulation (Fig. 1 and 2).

**TABLE 2:** MICs of resistant subpopulations emerging during treatment in the hollow-fiber infection model for isolate 1

Treatment	Subpopulation	MIC (mg/L) on treatment day 7 of:	
		Meropenem	Amikacin
Amikacin monotherapy	Amikacin resistant	4	64
	Meropenem resistant	32	64
	Amikacin and meropenem resistant		
Control (no treatment)	Amikacin resistant	2	64
	Meropenem resistant	32	64

**TABLE 3:** MICs of resistant subpopulations emerging during treatment in the hollow-fiber infection model for isolate 2

Treatment	Subpopulation	MIC (mg/L) on indicated treatment day of:			
		Meropenem		Amikacin	
		Day 3	Day 7	Day 3	Day 7
Amikacin monotherapy	Amikacin resistant	0.25	0.25	32	32
	Meropenem resistant	16	8	32	32
	Amikacin and meropenem resistant	4	8	64	64
Control (no treatment)	Amikacin resistant	0.5	0.5	32	32
	Meropenem resistant	4	8	4	4

**FIG 3:**
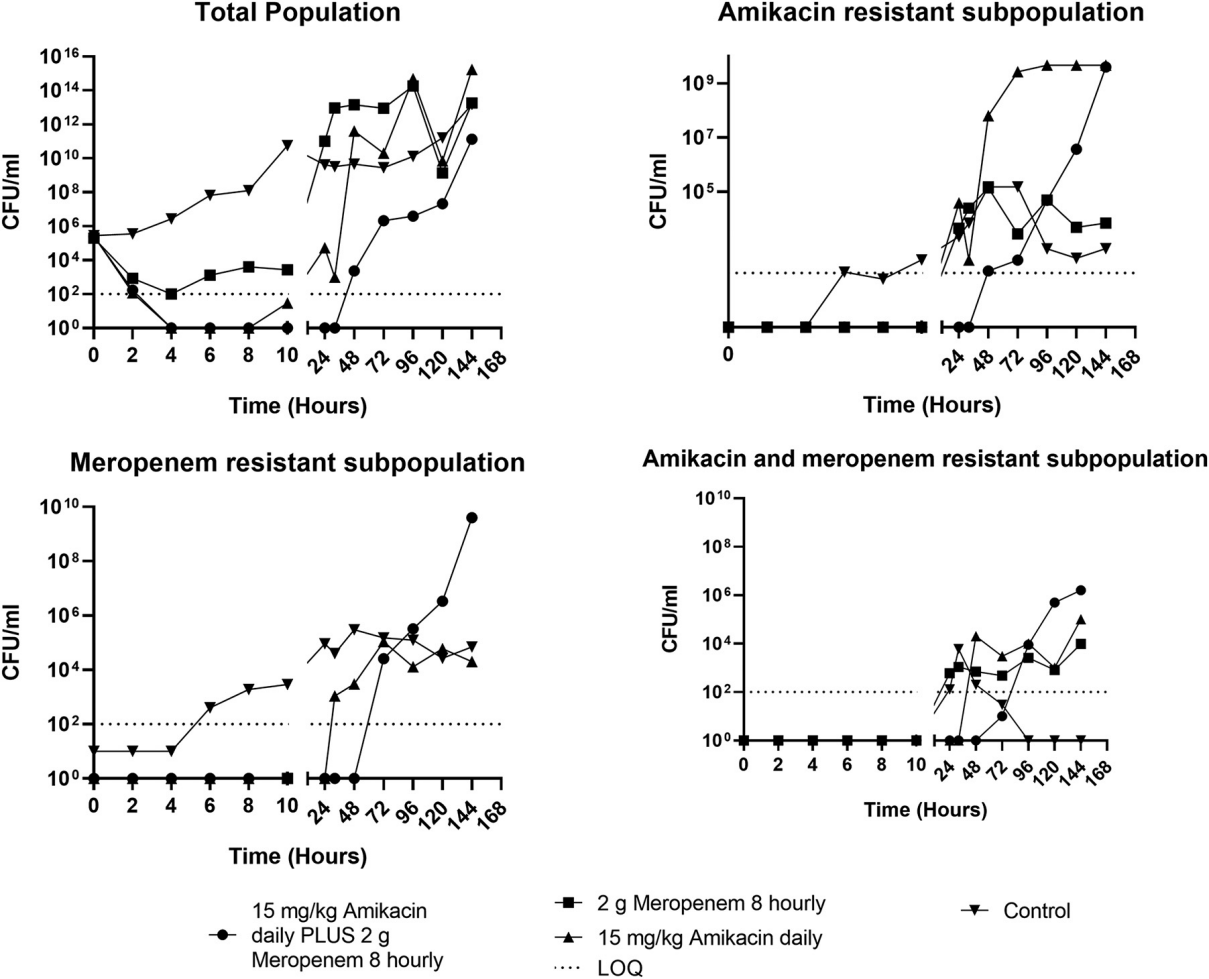
The effect of amikacin or meropenem monotherapy versus amikacin/meropenem combination therapy on the bacterial density of a P. aeruginosa isolate (isolate 3, P. aeruginosa isolate resistant to meropenem) in a hollow-fiber infection model. LOQ, limit of quantitation.

**FIG 4:**
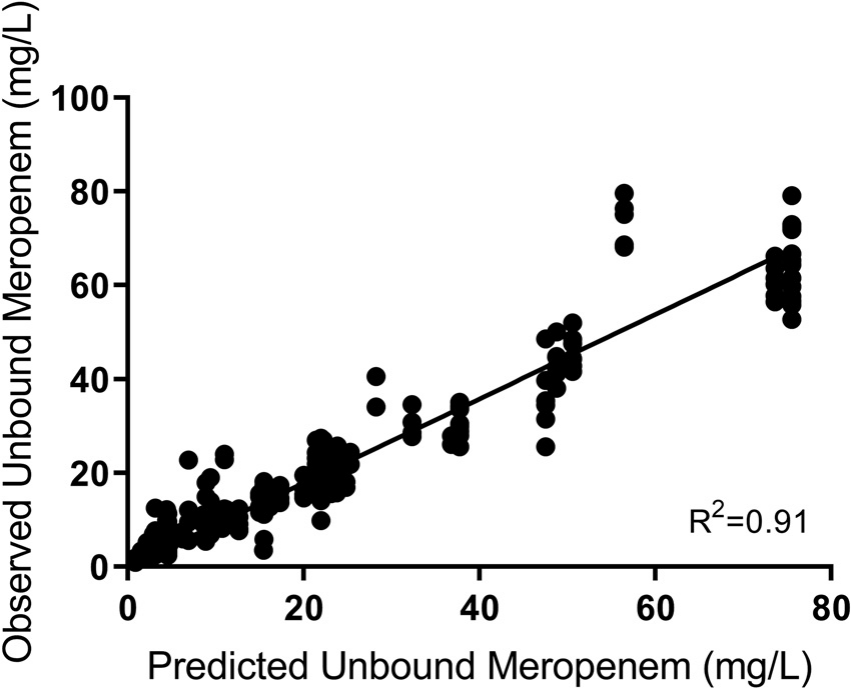
Observed versus expected concentration-time curve for meropenem.

**FIG 5:**
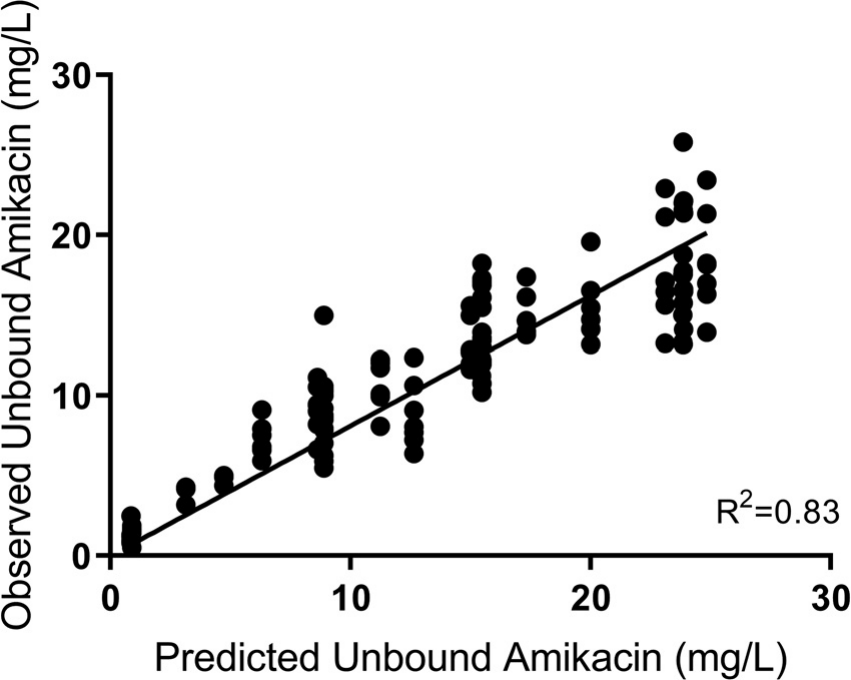
Observed versus expected concentration-time curve for amikacin.

For the isolate that was resistant to meropenem (isolate 3), monotherapy with amikacin and combination therapy with amikacin and meropenem reduced the bacterial density by ~5 log_10_ CFU/mL within the first 4 h of treatment (Fig. 3). However, monotherapy with amikacin was only able to sustain bacterial killing for the first 8 h of treatment, whereas the combination sustained bacterial killing for 32 h after initial dosing. Thereafter, rapid regrowth was observed, approximating the initial inoculum within 72 h (Fig. 3); regrowth mirrored growth on both amikacin- and meropenem-impregnated CaMH agar. Resistance to amikacin was confirmed with susceptibility testing, which showed that the MIC increased from 4 mg/L (Table 1) to 16 mg/L by day 7 of treatment (Table 4). The HFIM-observed meropenem (Fig. 4) and amikacin (Fig. 5) concentrations approximated the expected concentration-time curves.

**TABLE 4:** MIC testing was performed on isolates from each treatment arm and each type of agar plate isolated at the final time point for isolate 3

Treatment	Subpopulation	MIC (mg/L) on treatment day 7 of:	
		Meropenem	Amikacin
Amikacin monotherapy	Amikacin resistant	256	16
	Meropenem resistant	256	4
	Amikacin and meropenem resistant	256	16
Meropenem monotherapy	Amikacin resistant	128	16
	Amikacin and meropenem resistant	128	16
Amikacin and meropenem	Amikacin resistant	256	32
	Meropenem resistant	256	16
	Amikacin and meropenem resistant	256	16
Control (no treatment)	Amikacin resistant	256	4
	Meropenem resistant	256	4

## DISCUSSION

This study evaluated the impact of meropenem or amikacin monotherapy versus combination meropenem and amikacin therapy for clinical isolates of P. aeruginosa from bacteremic patients. Maximal bacterial killing was similar for both monotherapy and combination therapy for isolates susceptible to meropenem and amikacin, although the combination regimen increased the extent of bacterial killing by greater than 3 log (Fig. 1 to 3). In addition, the combination regimen and meropenem monotherapy were both able to sustain bacterial killing throughout the 7-day treatment course, whereas regrowth of bacteria occurred after 12 h with amikacin monotherapy. In contrast, for the P. aeruginosa isolate that was resistant to meropenem (but susceptible to amikacin), only the combination therapy was able to achieve extensive initial bacterial killing, although bacterial regrowth was evident after 32 h.

Overall, our findings are consistent with those reported previously for meropenem when used as monotherapy and when combined with an aminoglycoside (20, 21). A previous meropenem HFIM studying the bacterial killing against P. aeruginosa in simulated patients with a creatinine clearance of 120 mL/min receiving 2 g every 8 h also demonstrated a similar bacterial killing profile with sustained bacterial suppression (20). In the previous study, the *fC*_min/_MIC ratio was 2, lower than our own for susceptible isolates, which had an *fC*_min_/MIC ratio of >4. These findings are consistent throughout the literature, where an *fC*_min_/MIC ratio between 1 and 4 may suppress regrowth, whereas an *fC*_min_/MIC ratio of >4 commonly suppresses bacterial regrowth (21).

Our findings are also support a previous HFIM study simulating patients with augmented renal clearance receiving combined meropenem and tobramycin (22). Only the continuous infusion of meropenem and tobramycin was able to suppress regrowth for 7 days, even at a higher inoculum (~10^7^ CFU/mL) than was used in our study (~10^5^ CFU/mL) (22). This is a common finding in previous time-kill and pharmacokinetic/pharmacodynamic studies (23). The aminoglycoside enhances the beta-lactam antibiotic penetration, thereby increasing the concentration at the target site and associated bacterial killing (24). This would explain the findings of our study, where the combination of meropenem and amikacin increased the extent of bacterial killing against the meropenem-resistant isolate and even delayed regrowth compared with amikacin alone.

Our study also demonstrated regrowth of bacteria following amikacin monotherapy, with the emergence of subpopulations resistant to both amikacin and meropenem. This was most likely due to an amikacin-resistant subpopulation having existed in the initial inoculum. In addition, a subpopulation resistant to both amikacin and meropenem emerged with amikacin monotherapy, which suggested an amplification of the amikacin-resistant subpopulation and potential coselection of resistance. This has also been described in other HFIM studies, where susceptible bacteria were replaced with less-susceptible bacteria following amikacin monotherapy (20). Moreover, Drusano et al. described that the resistance mechanism specific for one drug had an impact on the required concentrations of both drugs to suppress the amplification of resistant subpopulations (25), suggesting that optimized amikacin dosing would be beneficial for these patients to reduce the risk that resistance will emerge. Although our study did not investigate the potential mechanism underlying the selection of resistance to both amikacin and meropenem following amikacin monotherapy, previous studies have shown aminoglycosides to induce MexAB efflux pump expression, leading to carbapenem resistance (26, 27).

The HFIM simulates a patient without an immune system (20). Patients who have the greatest ability to eradicate organisms like P. aeruginosa are those with a good host immune response, adequate source control, and appropriate antibiotic management (28). However, it is challenging to find a model best suited to optimize antibiotic management for those patients who are critically ill and have poor immune function, such as febrile neutropenic patients. Studies examining β-lactam antibiotics, including meropenem, undertaken in the HFIM correlate well with those from neutropenic mouse models and predict the emergence of resistance in patients (29, 30). Drusano et al. demonstrated for P. aeruginosa in a murine thigh infection that, while granulocytes contribute to the elimination of bacteria up to a certain level, this effect is saturable (31). Moreover, reducing the bacterial density to <1 × 10^2^ CFU/mL is a likely target for immunocompromised patients to reduce the probability of bacterial regrowth (31). Therefore, our results support the revised EUCAST recommendations of only using aminoglycosides as part of a combination regimen for systemic infections (12, 21). Therefore, the patients who are most likely to benefit from combination therapy are those who are immunocompromised or are likely to have a subtherapeutic β-lactam antibiotic exposure, such as those with augmented renal clearance or those infected with a higher-MIC isolate (32).

There are several strengths and limitations associated with our study. We were able to evaluate P. aeruginosa isolates that were both susceptible and resistant to meropenem to describe bacterial efficacy for monotherapy or combination therapy. As discussed previously, our HIFM model may be most applicable for an immunocompromised patient; however, characterizing antimicrobial exposures to optimize bacterial killing *in vitro* can aid clinical decision making regarding antibiotic dosing, potentially resulting in improved clinical outcomes. We tested conventional doses at only one creatinine clearance; therefore, the bacterial killing and emergence of resistance may be different with a higher or lower simulated antibiotic clearance, which would change drug exposures. We only simulated meropenem administered as an intermittent infusion. Previous HFIM studies have shown that the meropenem infusion method is critical for carbapenem-resistant P. aeruginosa (22), since only a continuous infusion of meropenem when combined with tobramycin suppressed bacterial regrowth. Additionally, we assessed only three isolates, and the bacterial killing efficacy and emergence of resistance may differ with other isolates.

The findings of our HFIM study against the tested isolates of P. aeruginosa support the initial use of combination therapy with meropenem and amikacin in critically ill or immunocompromised patients or in clinical settings where the probability of carbapenem resistance is high. In other clinical scenarios, with P. aeruginosa isolates that are susceptible to both meropenem and amikacin, our HFIM supports current meta-analyses that recommend β-lactam antibiotic monotherapy (6, 7). However, given the strain-specific pharmacodynamics of P. aeruginosa between the carbapenems and aminoglycosides, it difficult to exclude the possibility that combination therapy may be superior to monotherapy in all situations. Other investigators have demonstrated that the inclusion of a carbapenem in the combination was associated with improved survival if the meropenem MIC was 8 mg/L or lower (for which a high probability of attaining of the pharmacodynamic target exists if meropenem is used at high doses) (33, 34). Therefore, clinicians may want to consider using the combination therapy for the initial management and ceasing the aminoglycosides once antibiotic susceptibility results have been obtained, given the potential nephrotoxicity and vestibular and ototoxicity associated with this class of antibiotics (35).

Combination therapy using both amikacin and meropenem for the initial empirical management of P. aeruginosa infections offers some *in vitro* advantages over meropenem monotherapy, particularly for immunocompromised patients. Given that optimized dosing of individual antibiotics in combination can maximize potential synergy against some isolates, future studies should explore the conditions for a benefit of combination therapy against P. aeruginosa.

## MATERIALS AND METHODS

### Antimicrobial agents.

Analytical reference standards of meropenem (as trihydrate; Tokyo Chemical Industry Co. Ltd.) and amikacin (PHR1654; Sigma-Aldrich) were used for *in vitro* susceptibility testing. For dosing simulation in the hollow-fiber infection model experiments, clinical formulations of meropenem (meropenem trihydrate powder for injection; Ranbaxy Australia Pty. Ltd.) and amikacin (DBL Amikacin injection; Hospira Australia Pty. Ltd.) were used. For meropenem, a fresh 12.5-mg/mL dosing stock solution was prepared from the clinical formulation and kept as 1-mL aliquots in a −80°C freezer for preparation of the doses. For each meropenem dose, an aliquot was thawed immediately before dosing and the appropriate volume constituted with sterile broth, namely, CaMH (final volume, 20 mL) in a dosing syringe. Similarly, a 10-mg/mL amikacin dosing stock solution was prepared and stored in a refrigerator and an appropriate volume constituted with sterile broth (final volume, 20 mL) for dosing.

### Bacterial isolates.

Clinical isolates of P. aeruginosa from three patients were sourced from the University of Queensland Centre for Clinical Research. All bacteria were stored in CaMH containing 20% glycerol at −80°C. Prior to each experiment, fresh isolates were grown on Mueller-Hinton agar plates incubated at 37°C for 24 h and were used to prepare inocula. For the HFIM studies, the bacterial suspensions for inoculation were prepared from the freshly grown agar plates by first making a 0.5-McFarland standard suspension in sterile water and then constituting an appropriate volume into a 10-mL bacterial suspension in CaMH broth to achieve a starting inoculum of approximately 1 × 10^5^ CFU/mL to simulate a bloodstream infection. The culture was then incubated at 37°C for 12 h to give rise to 1 × 10^9^ CFU/mL based on a prior growth curve analysis. Finally, an appropriate aliquot of the 12-h culture was diluted with sterile broth (final volume 40 mL) to achieve a final inoculum concentration of approximately 1 × 10^5^ CFU/mL and subsequently confirmed by quantitative cultures. The bacterial burdens in the cultures were determined by plating the cultures and using a quantitative culture technique.

### *In vitro* susceptibility testing.

The broth microdilution method was used to determine the MICs of the organism in accordance with the recommendations of the Clinical and Laboratory Standards Institute (CLSI) and EUCAST (36, 37). In brief, serial 2-fold dilutions of each antibiotic were prepared in CaMH broth and aliquoted into round-bottom microtiter plates (CLSI) and flat-bottom microtiter plates (EUCAST). A standardized inoculum suspension prepared in CaMH broth was then added to give a final inoculum of ~5 × 10^5^ CFU/mL. The inoculated cultures were then incubated at 37°C for 16 to 20 h. The lowest modal concentration of the antibiotic that completely inhibited visible bacterial growth was identified as the MIC in accordance with the CLSI and EUCAST recommendations. The MIC tests were performed on two separate occasions, each with 4 replicates.

### Mutant frequency.

A 10-mL culture of a 10^2^ CFU/mL inoculum was incubated in CaMH broth for 24 h at 37°C. Quantitative culturing methods were performed on the resultant bacterial growth, using both drug-free CaMH agar plates and antibiotic-containing CaMH agar plates. The antibiotic concentrations for agar plates used in the mutant frequency study were one dilution above the breakpoint for susceptible isolates (Table 1), namely, 16 mg/L for meropenem and 32 mg/L for amikacin. For isolate 3, which was resistant to meropenem, the antibiotic concentration was two dilutions above the MIC (meropenem, 256 mg/L). The mutant frequency was taken as the ratio of the concentration of bacterial subpopulations growing on antibiotic-containing plates after incubating for 48 h at 37°C to the total bacterial concentration growing on drug-free agar plates.

### HFIM.

The circuit system for the hollow-fiber infection model (HFIM) was set up as previously described (38). FiberCell Systems cartridge C2011 was used for all experiments. In the experiments investigating the combination of meropenem and amikacin, a supplementing compartment was introduced to simulate the differential clearance of the two antibiotics, in accordance with the method described by Blaser (39).

The concentration-time profiles of meropenem and amikacin were simulated based on population pharmacokinetic models previously described by Mattioli et al. (40) and Romano et al. (41), respectively, assuming a patient weight of 80 kg and a creatinine clearance of 100 mL/min. This corresponded to simulated half-lives of 1.7 h and 4.8 h for meropenem and amikacin, respectively. The volume of the central compartment was set at 200 mL, and the corresponding systemic clearances calculated for meropenem and amikacin were 1.37 mL/min and 0.48 mL/min, respectively. The *fC*_max_ targets were 24.8, 42.6, and 85.2 mg/L for amikacin, meropenem 1 g, and meropenem 2 g, respectively. The targets for area under the concentration-time curve for the free, unbound fraction of the drug (*f*AUC) were 171.3, 96.5, and 193.6 mg · h/L for amikacin, meropenem 1 g, and meropenem 2 g, respectively.

Six separate circuit systems were set to simulate the clinical course of antibiotic therapy for the following regimens: (i) combination therapy with 15 mg/kg amikacin once daily plus 1 g meropenem 8-hourly; (ii) combination therapy with 15 mg/kg amikacin daily plus 2 g meropenem 8-hourly; (iii) monotherapy with 1 g meropenem 8-hourly; (iv) monotherapy with 2 g meropenem 8-hourly; (v) monotherapy with 15-mg/kg amikacin daily; and (vi) control (no drug therapy). All doses were administered as a bolus infusion over 30 min using a syringe pump, and the duration of treatment was 7 days. During the treatment period, serial bacterial samples were collected from the extracapillary space of the hollow-fiber bioreactor before the first dose and at 2, 6, 8, 10, 24, 32, 48, 72, 96, 120, 144, and 168 h after commencement of treatment. Each bacterial sample was washed twice with sterile phosphate-buffered saline. Appropriately diluted bacterial-suspension samples were plated on both antibiotic-containing and standard CaMH agar to quantify the likely resistant and total bacterial populations, respectively. The drug-containing CaMH agar plates included either meropenem, amikacin, or the combination of meropenem and amikacin at 4 times the baseline isolate MIC.

### Drug assay.

Amikacin and meropenem were measured in CaMH broth by a validated chromatographic method that was developed and validated in-house. For amikacin analysis, 30 μL of a CaMH broth sample was combined with tobramycin (internal standard) and acidified with trichloroacetic acid. An aliquot of the supernatant was injected onto a Nexera2 ultra-high-performance liquid chromatography (UHPLC) system coupled to an 8030+ triple quadrupole MS detector (Shimadzu, Kyoto, Japan). Chromatographic separation was achieved using a Luna omega polar C_18_ (1.6 μm), 50- by 2.1-mm analytical column (Phenomenex, Torrance, CA, USA) with an ammonium formate/formic acid/acetonitrile mobile phase. Detection of amikacin and tobramycin was performed using an electrospray source in positive mode with optimized multiple-reaction-monitoring (MRM) conditions for each analyte. Amikacin was monitored at MRM conditions of *m/z* 586.25 → 163.10, and tobramycin was monitored at *m/z* 468.20 → 162.95. For meropenem analysis, CaMH broth was directly injected onto a Nexera UHPLC system coupled to a photodiode array detector (Shimadzu, Kyoto, Japan). Meropenem was retained away from any interference on a Shim-pack XR-ODS III, 2.0- by 50-mm (1.6-μm) analytical column (Shimadzu, Kyoto, Japan) with a mobile phase of 87% phosphate buffer (0.1 M, pH 7) with 13% methanol. Meropenem was detected at 300 nm. The assay range for amikacin was 1 to 100 μg/mL and for meropenem was 0.5 to 200 μg/mL. The assay methods were validated for linearity, lower limit of quantification, precision, and accuracy according to both U.S. Food and Drug Administration (42) and European Medicines Evaluation Agency criteria. The precision levels were within 8.9% (amikacin) and 2.2% (meropenem), and the accuracies were within 12.8% (amikacin) and 6.2% (meropenem) at the concentrations tested (amikacin at 1, 3, 10, 40, and 80 μg/mL and meropenem at 0.5, 1.6, 16, and 160 μg/mL).
